# New options for targeting TRPV1 receptors for cancer treatment: odorous Chinese herbal medicine

**DOI:** 10.3389/fonc.2025.1488289

**Published:** 2025-02-11

**Authors:** Minghui Zhang, Zongao Wang, Shaojun Liu, Yuxuan Li, Yanting Gong, Min Liu

**Affiliations:** ^1^ Nanjing University of Chinese Medicine, Suzhou, China; ^2^ Suzhou TCM Hospital Affiliated to Nanjing University of Chinese Medicine, Suzhou, China

**Keywords:** TRPV1, cancer, tumor micro-environment, odorous traditional herbal medicines, apoptosis

## Abstract

Vanilloid1 (TRPV1), a subfamily of transient receptor channels, is one of the non-selective calcium channels, which is a bridge between cellular response and extracellular environmental networks, and is involved in a variety of pathophysiological processes. It is also involved in the process of cancer occurrence and progression, and researchers are revealing its role in cancer. In this paper, we review the expression and significance of TRPV1 receptor in various cancer cell types, the role of TRPV1 in the apoptosis-proliferation balance, cancer cell invasion and metastasis, and tumor micro-environment, with emphasis on the mechanisms by which TRPV1 receptor mediates inflammatory response, immune system, and thus regulates cancer. We discussed the latest directions and current challenges of TRPV1 receptor-targeting therapy for cancer, and summarized the odorous traditional herbs that modulate TRPV1 receptors, with a view to developing anti-tumor drugs targeting TRPV1 receptors in the future.

## 1 Introduction

According to the latest Global Cancer Statistics Report (1), there were nearly 20 million new cancer cases (including non-melanoma skin cancer) and 9.7 million deaths from cancer (including non-melanoma skin cancer) in 2022, cancer has become a public health problem that seriously jeopardizes the health of human beings. Most patients diagnosed with cancer are in the middle and late stage. However, among the treatment methods, surgical complications, radiotherapy and chemotherapy toxic side effects are large, which seriously affects the quality of life of patients. At present, the treatment of cancer is transitioning from traditional surgery, chemotherapy and radiotherapy to new immunotherapy, targeted therapy and ion channel therapy. Finding new targets or low-toxicity and high-efficiency drugs for cancer treatment has always been a hot field of medical research. TRPV1 has emerged as a potential target for cancer treatment due to its role in regulating tumor growth, cell apoptosis, and the tumor micro-environment.

Capsaicin receptor - transient receptor potential vanillate receptor (TRPV1) is a non-selective cation channel that can be activated by temperature, physical and chemical stimuli (2). Its activation regulates a variety of biological responses, such as apoptosis and proliferation (3), metabolism and glucose homeostasis control (4), as well as nociception and body temperature. Recent studies have shown that TRPV1 receptor plays an important role in tumor biology through a variety of pathways. However, their specific mechanisms and roles in different types of cancer are still not fully understood, and TRPV1 modulators still have a long way to go before becoming anti-tumor drugs due to their side effects. We believe that TRPV1 is a heat-activated channel that can be activated by some odorous traditional herbs. TRPV1 receptor is a popular target for cancer therapy, and these odorous traditional herbs may also be potential drugs for cancer prevention and treatment.

This review aims to summarize the mechanisms by which TRPV1 receptors influence cancer progression, including their role in tumor cell proliferation, apoptosis, invasion, and tumor micro-environment. In addition, the anti-tumor potential of odorous traditional herbs that regulate TRPV1 is also discussed, hoping to develop anti-tumor drugs targeting TRPV1 receptors in the future.

## 2 Overview of TRPV1 receptors

Transient receptor potential vanilloid receptor was the first member of the transient receptor potential cation channel family to be discovered, it was cloned from the rat dorsal root ganglion by Caterina et al. in 1997 (5), and later formally named TRPV1 (6). TRPV1 is a multi-modal injury receptor that can be activated or variably modulated by temperature, physical, and chemical stimuli (7). In addition to capsaicin, its thermal sensitivity is activated by other endogenous substances (ATP, bradykinin, nerve growth factor, inflammatory mediators such as arachidonic acid amide and prostaglandins) and exogenous physical or chemical stimuli (camphor, resinaceous toxins, vanilloid toxins 1-3, ginger, and ethanol) (8). Upon activation of the post-cation channel TRPV1, extracellular Ca^2+^ flows into cells and releases intracellular cisterna Ca^2+^, resulting in increased intracellular Ca^2+^ concentration and mediating various basic cellular activities, such as muscle cell contraction, neuronal activity and transmitter release, cell proliferation and apoptosis (9).

**Figure 1:**
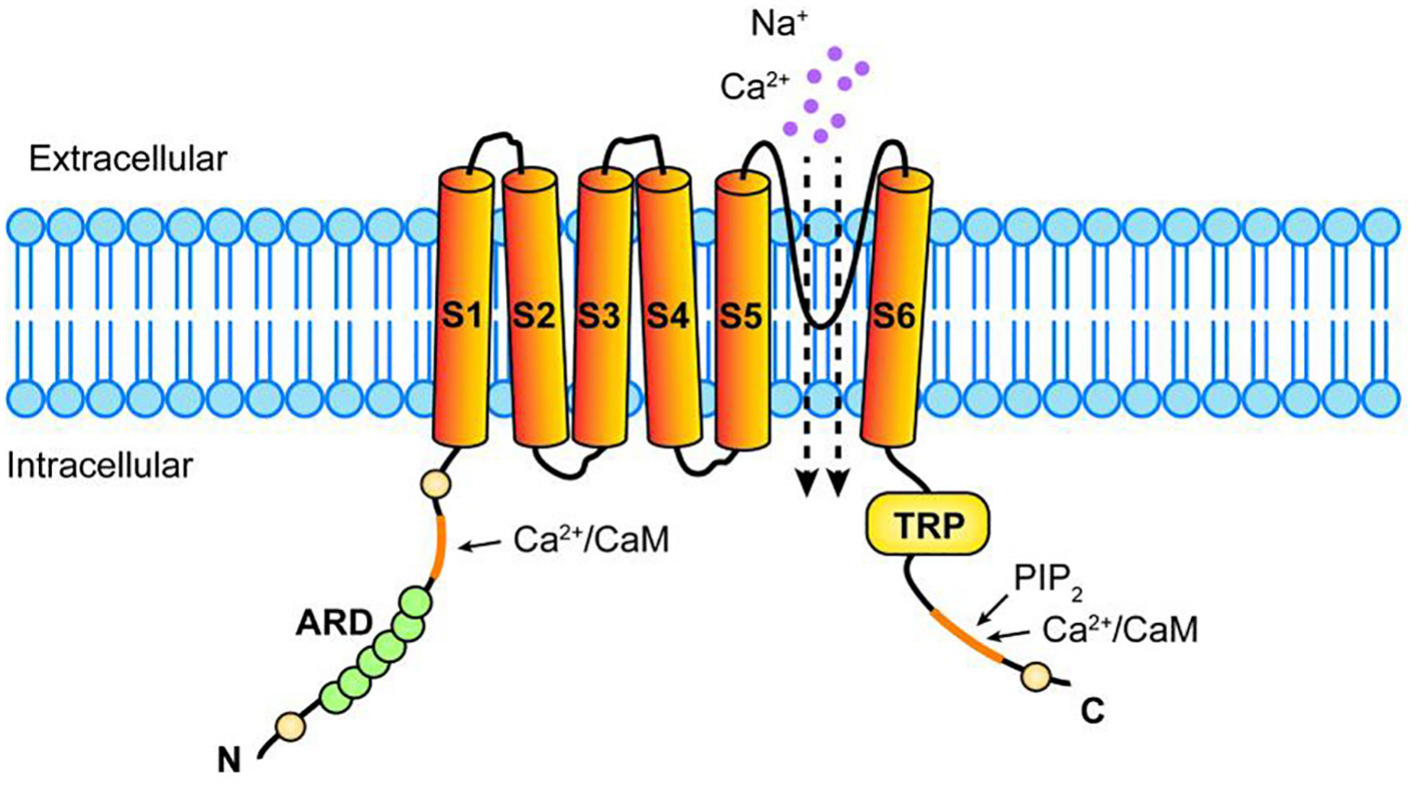
Structure of TRPV1 receptor. TRPV1 consists of four identical subunits assembled around a central hydrophobic pore, each consisting of six transmembrane domains (S1-S6), a long N-terminal region, and a relatively short C-terminal region. A hydrophobic pore mediates ionic passage between S5 and S6, N-terminal region has several phosphorylation sites and 6 ankyloprotein repeats (ARD) that bind calmodulin (CaM),The C-terminal region has TRP region, multiple CaM binding regions and endogenous substance binding sites, which can bind phosphatidylinositol-4,5 diphosphate (PIP2), Ca^2+^ and so on.

TRPV1 transmembrane protein is a homologous tetramer composed of four identical subunits assembled around a central water pore, each consisting of six transmembrane domains (S1-S6), a long N-terminal region, and a short C-terminal region (10, 11). There is a short hydrophobic hole between S5 and S6 that mediates the passage of ions; The N-terminal region contains several phosphorylation sites and six ankyloprotein repeats that bind calmodulin and ATP; The C-terminal region contains the TRP region, multiple calmodulin binding regions, and endogenous substance binding sites, which can bind protein kinase C (PKC), Ca^2+^/calmodulin-dependent protein kinase II (CaMKII), and phosphatidylinositol-4,5 diphosphate (PIP2) (12). Compound binding to these sites modulates TRPV1 sensitivity and function, we need to elucidate their ligand structures and corresponding TRPV1 sites, mechanisms of action in the cancer process, and final effects. (The structure of TRPV1 is shown in **Figure 1**).

## 3 TRPV1 receptor expression in cancer

TRPV1 mRNA and protein are expressed in many cancer cell lines, and the expression levels in different cancer tissues are significantly different from those in their normal tissues, suggesting that it is involved in key processes of cancer progression. Weber LV et al. found that the expression of TRPV1 in breast cancer tissues and cells was significantly higher than that of healthy breast tissues, and that the average expression level was the highest in the triple-negative breast cancer subtypes (13). TRPV1 expression in cervical cancer tissues was higher than that in precancerous epithelial tissues and normal epithelial tissues, and its up-regulated expression was significantly correlated with more advanced tumor stage and poorer tumor grading. Over-expression of TRPV1 significantly increased the proliferation and clonogenic ability of cervical cancer cells (14).

However, the expression level of TRPV1 in some cancer tissues was lower than that in their normal tissues. It was found that the expression of TRPV1 in melanoma tissues and cell lines was significantly lower than that in nevus tissues and normal melanocytes (15). The expression of TRPV1 was lower in cervical squamous cell carcinoma (CSCC) and cervical adenocarcinoma than in normal group, and lower in CSCC than in cervical adenocarcinoma group (16).

Apparently the expression level of TRPV1 in each cancer cell line is highly heterogeneous, and the expression of TRPV1 is associated with different tumor micro-environments and different cancer-associated pathways. The expression level of TRPV1 also changed in the same cancer tissue, which was related to the grade of cancer tissue, pathological stage and tumor stage. Therefore, the expression level of TRPV1 can be used for the diagnosis and prognosis of cancer. The changes in TRPV1 expression, the mechanism of change and its role depend on different cancer cell types, which still need further study. (Compared with normal tissues, the changes and significance of TRPV1 expression level in cancer tissues are shown in **Table 1**).

**Table 1:** Expression and significance of TRPV1 in cancer.

Type of Cancer	Changes in TRPV1 expression levels in cancer tissues compared to their normal tissues	Significance	Reference
11 breast cancer tissues and 49 breast cancer cell lines	Increase	Higher TRPV1 expression is associated with poorer prognosis	(13)
transitional cell carcinoma of human bladder(TCC)、Urethral cancer of human bladder(UC)	Decrease	Gradual loss of low TRPV1 expression suggests tumor progression, increased staging, higher malignancy, and worse prognosis.	(17)
human hepatocellular carcinoma (HCC)	Increase	TRPV1 has an anti-HCC progression effect and high expression is associated with a better prognosis in HCC patients.	(18)
LNCaP and PC-3 prostate cancer cells	Increase	–	(19)
gastric cancer	Decrease	Decreased expression of TRPV1 protein in GC tissues was positively correlated with tumor size, histological grade, lymphatic metastasis, and clinical stage.	(20)
Cervical Cancer	Increase	High expression of TRPV1 significantly enhances the proliferation and clonogenic ability of cervical cancer cells. TRPV1 in combination with PTEN is an effective prognostic marker for cervical cancer.	(16)
Melanoma	Decrease	TRPV1 is a potential tumor suppressor in melanoma, and the higher the expression, the better the prognosis.	(15)
Non-Small-Cell Lung Cancer、Adenocarcinoma of the lungs	Increase	TRPV1 expression promotes drug resistance in NSCLC. High TRPV1 expression is associated with poor prognosis in LUAD.	(21)
multiple myeloma	Increase	Higher TRPV1 expression is associated with poorer prognosis.	(22)
acute mononuclear cell leukemia	Increase	–	(23)
Glioblastoma multiforme (GBM)	Increase	TRPV1 was negatively correlated with grading and positively correlated with prolonged overall survival and had an inhibitory effect on GBN.	(24)
colorectal cancer	–	TRPV1 inhibits EGFR-induced proliferation of intestinal epithelial cells.	(25)
thyroid cancer	–	Activation of TRPV1 by capsaicin is associated with inhibition of metastasis in papillary thyroid carcinoma bCPAP cells.	(26)
endometrial cancer	Decrease	Lower TRPV1 expression is associated with worse prognosis.	(27)
pancreatic	Increase	–	(28)
esophageal squamous cell carcinoma cells	Increase	Higher TRPV1 expression is associated with poorer prognosis	(29)
Squamous cell carcinoma of the human tongue, mouth, floor of the mouth, gums, and head and neckOral	Increase	–	(30)
human renal carcinoma	Decrease	TRPV1 expression was not associated with the staging or grading of ccRCC; high TRPV1 expression predicted better OS and DFS in ccRCC	(31)
Human osteosarcoma	Increase	–	(32)

## 4 Role of TRPV1 receptor in cancer

### 4.1 Relationship between TRPV1 receptor and cancer cell proliferation and apoptosis

Intracellular Ca^2+^ is involved in the process of apoptosis and cell proliferation, and is a second messenger that influences the proliferation-apoptosis balance (33). Therefore, Ca^2+^ influx into the cytoplasm after activation of TRPV1 will change the balance between apoptosis and proliferation signaling pathways, which may promote the development of cancer and may also have anti-tumor effects.

After binding of exogenous agonists to TRPV1, the flow of Ca^2+^ from the cytoplasm into cells is a common step between apoptosis and proliferation pathways. TRPV1 activation passes through the mitochondrial pathway, the endoplasmic reticulum pathway, and the exogenous death receptor pathway, ultimately leading to activation of the protease caspase 3, which mediates apoptosis through nuclear activity (34). The mechanism by which TRPV1 promotes cell proliferation is still under investigation, and two mechanisms have been derived: (1) TRPV1 activation causes ATP to be released into the extracellular space, where ATP binds to the G-protein-coupled receptor P2Y2, initiating a kinase signaling cascade that activates serine/threonine kinase (Akt); (2) TRPV1 trans-activates the EGFR, which results in the activation of a series of protein signals that activate extracellular signal-regulated kinase1/2 (ERK1/2). Ultimately Akt and ERK 1/2 MAPK promote cell proliferation through nuclear activity (35).

The activation of TRPV1 affects apoptosis or proliferation through different and competitive pathways, which may explain why TRPV1 is differently expressed in different types of cancer and the reason and effect of the expression change. The higher expression of TRPV1 in more malignant cancer cell lines may be due to the fact that it mainly promotes cell proliferation; On the contrary, the lower expression of TRPV1 in the more malignant cancer cell lines may be due to the fact that it mainly plays a role in promoting apoptosis. Therefore, it is promising to regulate TRPV1 to restore the balance of the cell proliferation-apoptosis signaling pathway to achieve anti-cancer effects. For example, TRPV1 was found to be over-expressed in intestinal epithelial HCT116 cells, and TRPV1 inhibited EGFR-induced proliferation of epithelial cells through activation of Ca^2+^/calpain and protein tyrosine phosphatase 1B (PTP1B) (25). Over expression of TRPV1 or the action of its agonist capsaicin can inhibit melanoma growth by activating p53 and inducing apoptosis (15).

TRPV1 also regulates the apoptosis-proliferation balance through mechanisms other than Ca^2+^ signaling, which may be related to the interaction of cellular receptors and cytokines in the tumor micro-environment (35), it may also be related to the type of agonist, the type of cancer, and the inflammatory response. Therefore, TRPV1 may play a multi-directional role in cancer tissue.

### 4.2 TRPV1 receptors inhibit the invasion and metastasis of cancer cells

Cancer invasive metastasis is an important cause of mortality in malignant tumors, matrix metalloproteinases (MMPs) promote cancer cell migration by degrading extracellular matrix (ECM), basement membrane (BM) and remodeling intercellular adhesion. It can promote epithelial-mesenchymal transition (EMT), apoptosis of anticancer cells and promote angiogenesis in tumor micro-environment. Currently, It has been found that TRPV1 receptor can inhibit the invasion and metastasis of cancer cells by regulating MMPs (36). XuS et al. (26) found that capsaicin activation of TRPV1 down-regulates the expression of key EMT transcription factors Snail1 and Twist1, and also down-regulates the expression of MMPs-2 and MMPs-9, and ultimately significantly inhibits the migration, invasion and adhesion of bCPAP cells in thyroid papillary carcinoma. Robert Ramer et al. (37) found that cannabidiol up-regulated intercellular adhesion molecule-1 through cannabinoid receptor, TRPV1 receptor, and p42/44 mitogen-activated protein kinase in lung cancer cell lines A549, H358, and H460, inhibiting lung cancer cell invasion. Nuray Erin et al. (38) found that the TRPV1 agonist olvanil activated TRPV1-containing sensory nerve fibers, enhanced T-cell responses, and significantly inhibited breast cancer metastasis. This anti-breast cancer metastatic effect may be mediated through the neuroimmune pathway. In fact, MMPs are also involved in the immune response, and perhaps it plays a role in TRPV1’s anti-breast cancer metastasis through the neuroimmune pathway, which needs to be further investigated.

In addition, We need to pay attention to the fact that TRPV1 regulators can inhibit cancer cell migration and invasion through non-TRPV1 pathways. For example, in bladder cancer cells (39), Capsaicin reduces the deacetylase of SIRT1, enhances the acetylation of cortactin and β-catenin, thereby reducing the activation of MMP-2 and MMP-9, and ultimately leads to the migration disturbance of bladder cancer cells.

### 4.3 TRPV1 receptor is associated with tumor micro-environment

**Figure 2:**
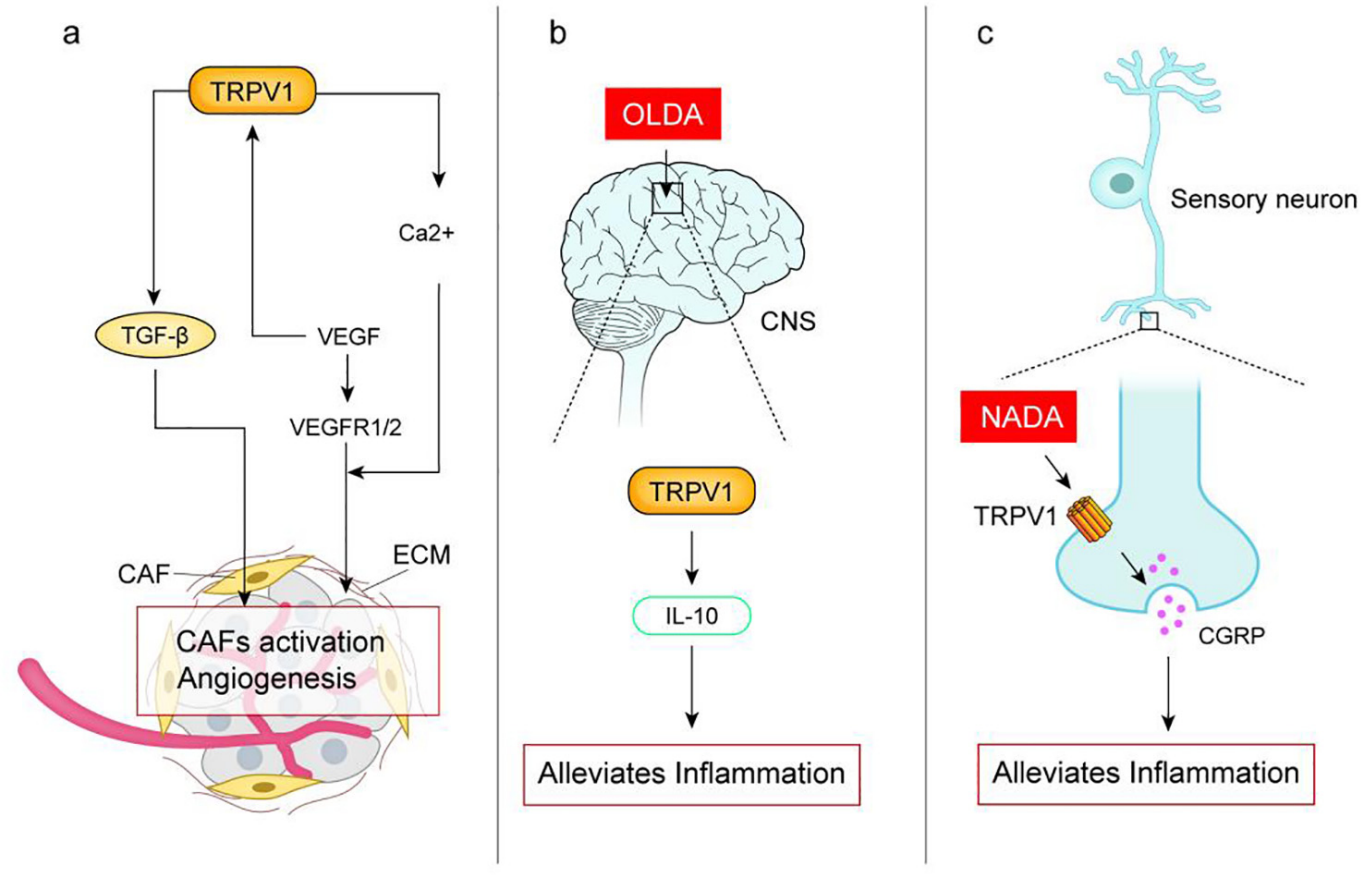
**(A)** Activation of TRPV1 upregulates the expression of transforming growth factor TGF-β, which recruits and activates cancer-associated fibroblasts (CAF), which regulate the formation of ECM ands promote tumor proliferation and metastasis; Vascular endothelial growth factor (VEGF) secreted by human uveal melanoma cells activates TRPV1 leading to intracellular Ca2+ inward flow, which is essential for angiogenesis. VEGF binds to VEGFR1/2 can induce proliferation and migration of vascular endothelial cells and promote angiogenesis. **(B)** TRPV1 endogenous agonist N-oleoyldopamine (OLDA) reduces inflammatory response by activating the central nervous system TRPV1 induces IL-10; **(C)** TRPV1 endogenous agonist N-dopamine (NADA) activates peripheral sensory neurons TRPV1 inhibits the release of calcitonin gene-related peptide (CGRP) thereby reducing acute inflammation. Studies have shown the pro-inflammatory effects of TRPV1, which may be related to the fact that activation of TRPV1 in different tissues produces different effects, or may be related to the type of agonist.

Tumor is not only a simple collection of cancer cells, but also constitutes the tumor micro-environment (TME) with its internal and external environment. TME is mainly composed of tumor Extracellular matrix (ECM), Tumor vascular system, Cancer-associated fibroblasts (CAF), immune system and inflammation, which jointly affect the occurrence and development of tumors (40). There is an association between TRPV1 and the tumor micro-environment (The assciation is shown in **Figure 2**).

#### 4.3.1 TRPV1 receptor and cancer-associated fibroblasts

TRPV1 may regulate TGF-β/CAF and influence cancer development. CAF are a major component of the ECM, which regulates ECM formation and promotes tumor proliferation and metastasis (41). TGF-β, a transforming growth factor secreted by tumors, recruits and activates CAFs. Bodo et al. (42) demonstrated that capsaicin activation of TRPV1 up-regulated the expression of TGF-β2 mRNA and protein in human hair follicles, but the mechanism remains unclear. In TRPV1 knockout animal models, TGF-β1 expression was attenuated (43). These suggest that TRPV1 may regulate transforming growth factor TGF-β, modulating CAF, and thus be involved in cancer development.

#### 4.3.2 TRPV1 receptor and tumor vascular system

TRPV1 is involved in tumor angiogenesis by regulating the expression and function of VEGF and its receptors. Tumor vascular system is a key process in tumor growth and metastasis, tumor cells or ECM can release key proteins vascular endothelial growth factor (VEGF) to bind to VEGFR1/2, induce vascular endothelial cell proliferation and migration, and promote angiogenesis (44). VEGF secreted by human uveal melanoma cells was found to activate TRPV1 function, leading to intracellular Ca^2+^ endocytosis to endothelial cells, and intracellular Ca^2+^ endocytosis is required for angiogenesis (45). Studies have shown that in TRPV1-/- mice, the NF-kB and STAT3 signaling pathways are overactivated, leading to a group of inflammatory factors (including IL-1 and IL-6) as well as invasive factors (such as MMP9 upregulation) that contribute to the development of cancer (46). NF-kB and STAT3 are known to be regulators of VEGF in several tumors (47) and may be involved in the regulation of VEGF expression by TRPV1. However, TRPV1 regulates the expression and function of VEGF and its receptors and is involved in tumor angiogenesis process, which is still uncertain and needs further study.

#### 4.3.3 TRPV1 receptor and inflammation, neuroimmune pathways and immune cell function

TRPV1 is expressed not only in cancer cells, but also in sensory neurons, the central nervous system, and a variety of immune cells (lymphocytes, dendritic cells, macrophages, and neutrophils). It is involved in the cancer process by regulating inflammatory responses, neuroimmune pathways, and immune cell function.

According to the current study, TRPV1 has a contradictory role in the inflammatory process. TRPV1 was found to be pro-inflammatory: TRPV1 is over-expressed in inflamed tissues (48), the use of TRPV1 antagonists or gene ablation attenuates the inflammatory response (49); TRPV1-expressing peripheral sensory neurons produce and release pro-inflammatory neuropeptides from their peripheral terminals, such as substance P (SP) and calcitonin gene-related peptide (CGRP), which subsequently cause neurogenic inflammation promoting cancer progression (50).

However, TRPV1 also has anti-inflammatory effects. Its activation may secrete neuropeptides with anti-inflammatory properties such as growth inhibitors (51). TRPV1 endogenous agonists such as N-oleoyldopamine (OLDA) induce IL-10 by activating TRPV1 in the central nervous system to reduce inflammatory responses and improve endotoxemia and sepsis outcomes (52). N-dopamine (NADA) reduces acute inflammation by activating TRPV1 (53). TRPV1 modulation of inflammation occurs at multiple levels and is associated with many regulatory proteins and inflammatory mediators, which leads to different final outcomes. Ca^2+^ plays a key role in the activation, differentiation, proliferation, cytokine secretion and effector functions of immune cells. TRPV1 acts as a calcium channel to regulate the function of immune cells (54). TRPV1 has been associated with macrophage-induced defense mechanisms. Activation of TRPV1 by Evodiamine or Capsaicin in mouse macrophages attenuates the inflammatory response induced by TNF-α, while CPZ, a TRPV1 antagonist, eliminated this anti-inflammatory effect (55).

Dendritic cells (DCs) are cells of the innate immune system responsible for antigen presentation, stimulation of T cells, and shaping the adaptive immune system. Activation of TRPV1 by Capsaicin stimulated the maturation and migration of DCs to lymph nodes in mice. In addition, TRPV1 was found to induce the release of calcitonin gene-related peptide (CGRP), a neuropeptide that regulates DC activation and T helper cell type 1 polarization (56). In the context of inflammatory and autoimmune diseases, TRPV1 has a broader role in T cells, where it is involved in TCR signaling, T cell proliferation and differentiation, and cytokine production (57).

TRPV1 activation may be important for maintaining the dynamic balance of immunity, however the exact molecular pathways by which TRPV1 regulates inflammation and immunity need to be further investigated. In conclusion, from the point of view of targeting TME and utilizing the immune system against tumors, TRPV1 receptor is a good choice.

## 5 TRPV1 receptor and cancer therapy

TRPV1 receptor is a popular target for the development of anti-cancer drugs. Pharmacological modulators of specific TRPV1 channels may promote or induce cancer cell death, and may also be effective in reducing cancer cell proliferation, invasion, and metastasis. In addition, it can achieve anti-cancer effects by regulating the tumor micro-environment. TRPV1 modulators can enhance the effect of chemoradiotherapy, and the synergistic use of TRPV1 modulator can reduce the required dose of chemoradiotherapy to control cancer and reduce the toxic side effects of chemoradiotherapy. TRPV1 agonist combined with chemoradiotherapy is a feasible strategy for cancer treatment (58). However, the systemic use of TRPV1 modulators causes side effects such as hyperthermia, burns and respiratory injury (59), which severely limits its application. Only low-concentration cream or transdermal patch types of topical analgesic preparations are currently in clinical use (60). The key point is how to ensure the anti-cancer effect of TRPV1 modulators while overcoming their side effects. Some studies have combined TRPV1 modulators with the latest drug delivery systems in the hope that precise localized drug delivery can avoid the activation of systemic TRPV1 receptors (61, 62); Others have been devoted to the development of new structural variants of TRPV1 modulators (63). However, all the current studies are still in the experimental stage, and there is still a long way to go before practical clinical application.

We may be able to turn our attention to the potential of traditional herbs, which traditional Chinese medicine categorizes as odorous traditional herbs that elicit odorous flavors and heat sensations, and use them to treat tumors (64, 65). TRPV1 receptors are activated by a number of irritating substances and temperatures above 43°C. Studies have shown that many odorous traditional herbs activate the TRPV1 receptor. The efficacy of odorous traditional herbs is highly similar to the physiological functions mediated by TRPV1 receptors, such as dispelling cold and relieving pain (66). Capsaicin, an extract of odorous traditional herbs, activates the TRPV1 receptor against tumors (67). Gingerol, an extract of ginger (68), has anti-tumor effects, studies have shown that gingerol can activate TRPV1 (69), but no one has studied whether it is anti-tumor through the TRPV1 receptor pathway. Curcumin, an extract of turmeric, also has anti-tumor effects (70), studies have shown that curcumin can antagonize TRPV1 receptors (71). The TRPV1 receptor is a popular target for treating cancer, We hypothesize that these odorous herbs can modulate the TRPV1 receptor to exert anti-tumor effects, which needs further research and may be a more promising direction for finding anti-cancer drugs that target TRPV1 receptors. We summarize the currently known herbs that regulate TRPV1 receptors, as shown in **Table 2**:

**Table 2:** Odorous herbs currently known to reglatory TRPV1 receptors.

odorous herb	Active compound	Chemical properties	Function	Antitumor Mechanism
Capsicum	Capsaicin	Vanillin	Agonist (67)	
Ginger、 Galangal (Zingiberaceae)	Gingerol	Guaiacols	Agonist (69)	(68)
	Shogaol、 Zingerone	Phenols		
	Paradol			
Peppercorn	Piperine	Alkaloids	Agonist (72)	(73)
Fructus evodiae	Evodiamine		Agonist (74)	(75)
Garlic	Allicin	Thiosulfinates	Agonist (76)	(77)
Clove	Eugenol	Allylbenzenes	Agonist (78)	(79)
Angelica 、Fructus cnidii	Imperatorin	Coumarins	Agonist (80)	(81)
Mugwort	Eugenol	Allylbenzenes	Agonist (82)	(79)
Asarum sieboldii Miq	Asarinin	–	Agonist (83)	(84)
Turmeric、 Curcuma Zedoaria、 Curcuma Longa(Curcuma L)	Curcumin	Phenolic antioxidants	Antagonism (71)	(70)
Monkshood	Aconitine	Diterpenoid alkaloids	Antagonism (85)	(86)

## 6 Conclusion and perspective

TRPV1 is a multifunctional cell receptor that is widely distributed in human tissues and can sense various stimuli and be converted into calcium-based cascades, a variety of physiological and pathological processes based on it are being discovered. TRPV1 receptors are involved in the development of cancer in various ways, and the specific mechanisms have not been fully elucidated. The occurrence of tumor is a multi-factor, multi-step, complex and long biological process. In the process of development, the uneven distribution and effects of environmental factors and the randomness of gene mutations lead to high heterogeneity of tumors. TRPV1 receptor is also affected by these factors in the occurrence and development of various types of cancer, activating different factors and molecular pathways to produce different effects. Therefore, activation of TRPV1 has different effects on different types of cancers, which needs to be further investigated.

The TRPV1 receptor is a biomarker for predicting and diagnosing cancer, Its expression levels in cancer tissues are highly heterogeneous. It is necessary to study its progression in specific cancer types, determine the range of its expression levels and the relationship between its changes and prognosis. A recent study (24) revealed for the first time the expression, clinical value and potential mechanism of TRPV1 in pancarcinoma, which is also a direction.

In terms of the regulation of the apoptosis-proliferative balance of cancer cells and the tumor micro-environment, we need to consider that TRPV1 receptor is involved in the cancer process in multiple ways, the results of the studies may be different, which is the reason why many studies have conflicting results. When designing relevant experiments, we should carefully consider the type of cancer cell, the type of experiment (*in vivo* or *in vitro*), the dose, concentration and duration of action of TRPV1 modulator, etc. We also need to circumvent the effects of the non-TRPV1 pathway of TRPV1 modulators on cancer and the effects of other TRPV1 cancer pathways so that the experimental results can be accurate. At present, the research on the role of TRPV1 in cancer is still in the initial stage. How to control multiple confounding factors in the experiment to make the results accurate is the current problem. In addition, it needs to overcome its serious side effects in humans.

There is no doubt that manipulating the TRPV1 receptor could treat cancer, and the possibilities are endless. We think it is very promising to find suitable medicines from traditional herbs, because traditional herbs have been practiced for thousands of years and have a safe dose range, they can activate TRPV1 receptors while avoiding side effects. We summarized the currently known odorous herbs that can regulate TRPV1 receptors, hoping to develop anti-cancer drugs targeting TRPV1 receptors in the future.
